# 420. Prevalence of comorbidities and COVID-19 vaccination among COVID-19 deaths

**DOI:** 10.1093/ofid/ofad500.490

**Published:** 2023-11-27

**Authors:** Zin Lyons, Lauren DiBiase, Emily Sickbert-Bennett, David J Weber

**Affiliations:** UNC Medical Center, Chapel Hill, North Carolina; UNC Health Care, Chapel Hill, NC; UNC Medical Center, Chapel Hill, North Carolina; University of North Carolina, Chapel Hill, NC

## Abstract

**Background:**

It is crucial to note that comorbidities may significantly increase the risk of mortality among COVID-19 patients. That's why it's vital for individuals with multiple comorbidities to make getting fully vaccinated a top priority, in order to safeguard their own health and the health of those around them. This study describes prevalence of comorbidities and COVID-19 vaccination among patients who died of COVID-19.

**Methods:**

This was a retrospective study that assessed 220 inpatients at the UNC Medical Center who died with COVID-19 per discharge diagnosis from 7/11/21-4/3/23. Data included demographics, COVID-19 vaccination status and preexisting comorbidities. Comorbidities were classified based on CDC designated risk factors for severe COVID-19 (CDC, 2023).

**Results:**

Of 220 patients who died, 45% were > 65 years, 60% were male, and 90% of patients had comorbidities ranging from 1 to > 3. The top 6 comorbidities among all genders and ages were hypertension (48%), heart conditions (42%), diabetes (35%), chronic kidney disease (29%), immunocompromised (29%), and obesity (25%), respectively. The proportion vaccinated was 38%; among those vaccinated, doses received were 1 (11%), 2 (40%), 3 (32%), 4 (10%), and 5 (7%). Among those who died and were vaccinated, they were 1.13 times more likely to have comorbidities (97%) compared to those not vaccinated (86%) (Risk ratio (RR) 1.13, 95% CI 1.05-1.22, P=0.004) (Table).

Prevalence of comorbidities and vaccination among COVID-19 deaths

**Figure:**
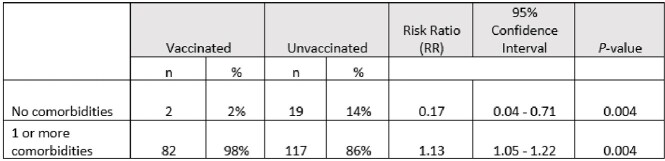

**Figure:**
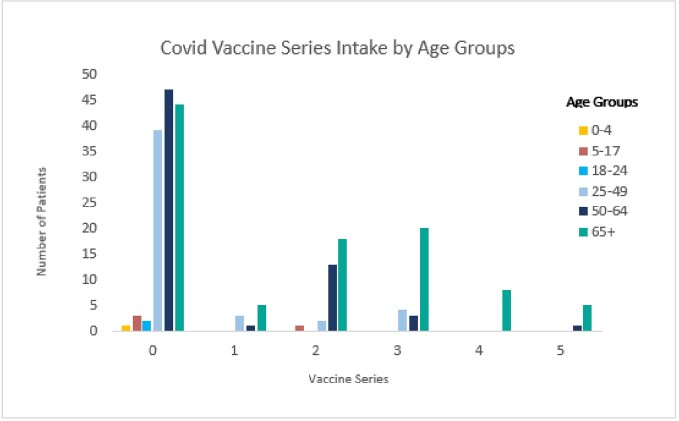

**Conclusion:**

In this cohort study of COVID-19 deaths, the majority of patients dying with COVID-19 were White, non-Hispanic, males > 65 years with comorbidities, most often with multiple comorbidities. Hypertension, heart conditions, diabetes, chronic kidney disease, immunocompromised, and obesity emerged as the most common comorbidities. More than half of the patients had not received COVID-19 vaccination. This highlights the importance of getting fully vaccinated, especially for those with pre-existing conditions.

**Disclosures:**

**David J. Weber, MD, MPH**, BD: Advisor/Consultant|Germitic: Advisor/Consultant|GSK: DSMB|PDI: Advisor/Consultant|Pfizer: Advisor/Consultant|Wellair: Advisor/Consultant

